# Successful embolization of mesenteric hematoma following blunt trauma: A case report

**DOI:** 10.1016/j.radcr.2025.04.015

**Published:** 2025-05-03

**Authors:** Rika Yoshida, Hisatoshi Araki, Yuki Komatsubara, Yuka Ishikura, Mitsunari Maruyama, Shinji Ando, Megumi Nakamura, Takeshi Yoshizako, Akihiko Kidani, Yasushi Kaji

**Affiliations:** a Department of Radiology, Shimane University, Faculty of Medicine, Izumo, Shimane, Japan; b Department of Radiology, Masuda Red Cross Hospital, Masuda, Shimane, Japan; c Department of Acute Care Surgery, Shimane University, Faculty of Medicine, Izumo, Shimane, Japan

**Keywords:** Mesenteric hematoma, Blunt trauma, Transcatheter arterial embolization, Minimally invasive, Bowel ischemia

## Abstract

Mesenteric injuries are rare, occurring in approximately 1% of all blunt trauma cases. Herein, we report the case of a man in his 70s who sustained an isolated mesenteric hematoma following blunt abdominal trauma from a detached tire. The hematoma was successfully managed with transcatheter arterial embolization (TAE), avoiding the need for invasive surgery and preventing bowel ischemia. This case highlights the importance of early diagnosis and prompt intervention with TAE in hemodynamically stable patients with mesenteric injuries.

## Introduction

Mesenteric injuries are uncommon but potentially life-threatening complications of blunt abdominal trauma [[1], [2], [3], [4], [5], [6]]. These injuries can lead to bowel ischemia, necrosis, peritonitis, and sepsis, emphasizing the need for early diagnosis and effective management. Isolated mesenteric hematoma, a condition in which blood accumulates in the mesentery without bowel perforation, is particularly challenging to manage. Although conservative treatment is often preferred, transcatheter arterial embolization (TAE) has emerged as a valuable minimally invasive treatment option for controlling bleeding and preventing complications.

### Case report

A man in his seventies was struck by a detached rear tire from a truck while walking. He was airlifted to our hospital. The patient reported no known allergies or family medical history. His past medical history includes hypertension (treated with antihypertensive medication) and lumbar disc herniation (history of 2 surgeries at another hospital).

The primary survey revealed no immediate life-threatening injuries. Upon arrival, the airway was patent, and no respiratory distress was observed. The radial pulse was palpable, with a capillary refill time of 1 second. Continuous bleeding from the face was noted. Glasgow Coma Scale score was 13, with Eye (E) 3: opens eyes to voice; Verbal (V) 4: Confused conversation; and Motor (M) 6: obeys commands. The patient’s vital signs were as follows: heart rate, 98 beats per min; blood pressure, 123/102 mmHg; respiratory rate, 16 breaths per min; and oxygen saturation, 97% on a reservoir face mask at 8 L/min. The patient’s arterial blood gas analysis indicated acidosis, with a pH of 7.327 (normal range, 7.35-7.45). The partial pressure of carbon dioxide (PaCO2) was 44.1 mmHg (normal range, 35.0-48.0), which is within the normal range. However, the partial pressure of oxygen (PaO2) was significantly elevated at 195.0 mmHg (normal range, 83.0-108.0). The base excess was −3.0 mmol/L (normal range, −3 to 3), also within the normal range. Notably, the lactate level was elevated at 17.0 mg/dL (normal range, 4.5-13.5). These findings indicate compensated metabolic acidosis, which is likely influenced by supplemental oxygenation and hyperlactatemia.

The second survey revealed multiple fractures (orbital bones, pelvis, and metacarpals) and bilateral pulmonary confusion.

Initial laboratory investigations revealed leukocytosis (white blood cell count, 14.37 × 10^3^/μL; normal range, 3.30–8.60 × 10^3^/μL); anemia (hemoglobin, 12.1 g/dL; normal range, 13.7-16.8 g/dL; hematocrit, 35.4%; normal range, 40.7%-50.1%); and thrombocytopenia (platelet count, 213 × 10^3^/μL; normal range, 158–348 × 10^3^/μL). The coagulation studies were also abnormal, with an elevated international normalized ratio of 1.08 (normal range, 0.90-1.10), low fibrinogen level of 184 mg/dL (normal range, 200-400 mg/dL), and elevated D-dimer level of 74.0 μg/mL (normal range, ≤1.0 μg/mL).

**Fig. 1:**
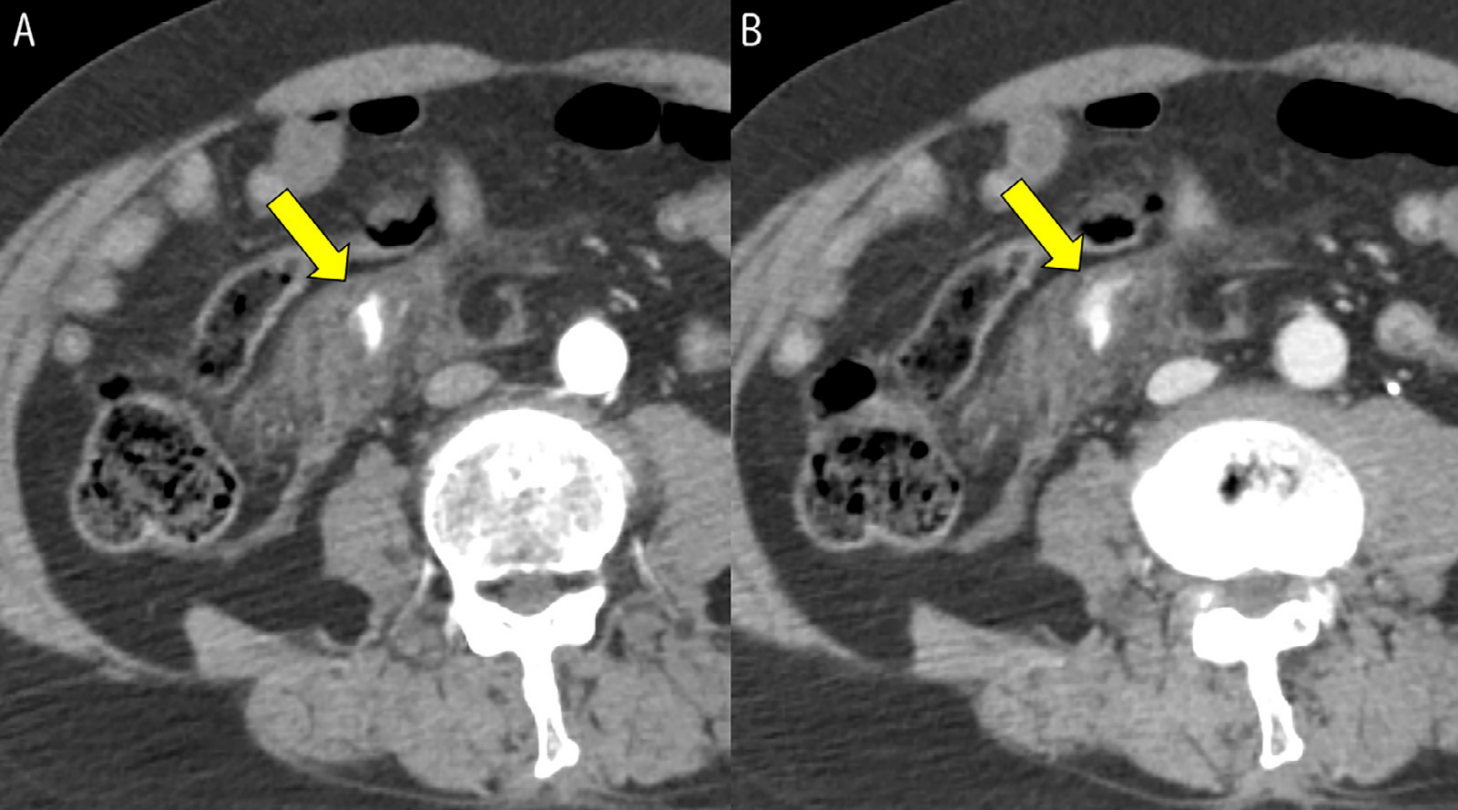
Abdominal contrast-enhanced CT. (A) arterial phase; (B) equilibrium phase (B is the same level as A) Contrast-enhanced computed tomography (CT) revealed an isolated mesenteric hematoma with contrast extravasation in the territory of the ileocolic artery in the right lower abdomen during the arterial phase (A, arrow). The contrast material spread further in the equilibrium phase (B, arrow).

A head computed tomography (CT) scan revealed traumatic subarachnoid hemorrhage and acute subdural hematoma. Abdominal contrast-enhanced CT revealed an isolated mesenteric hematoma (Fig. 1), pelvic fracture, and retroperitoneal hematoma; however, there was no evidence of bowel ischemia or perforation. Considering the patient’s stable vital signs and absence of an immediate surgical indication, we opted for management with TAE to achieve hemostasis.

**Fig. 2:**
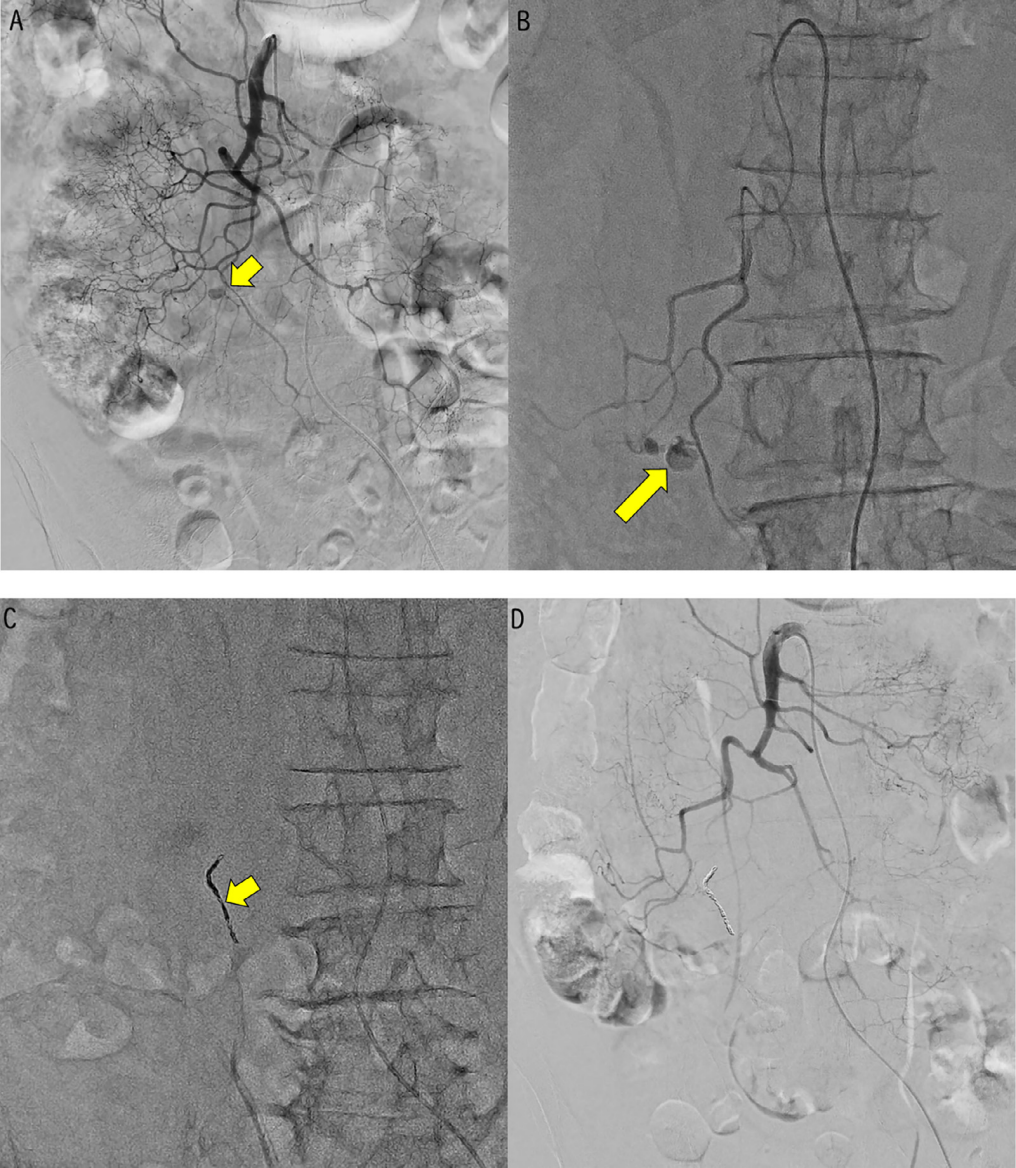
Angiography. (A) Selective digital subtraction angiography (DSA) of the superior mesenteric artery (SMA). (B) Selective digital angiography of the ileocolic artery. (C) Coil status after embolization on X-ray. (D) Postembolization DSA. Selective angiography of the SMA revealed extravasation from a branch of the ileocolic artery (A, B, arrow). Coil embolization was performed using detachable microcoils (C, arrow). Postembolization DSA confirmed successful hemostasis at the site of injury, with no evidence of active extravasation. Collateral blood flow to the terminal ileum and ascending colon was preserved via the marginal arteries (D).

Angiography revealed a vascular wall injury in the branch of the ileocolic artery (Fig. 2). The procedural and material details are described below. Access was gained through the left common femoral artery using a 4 French (Fr) sheath (Super Sheath®; Medikit, Tokyo, Japan). Superior mesenteric angiography revealed vascular wall injury of a single branch of the ileocolic artery. Selective catheterization of this branch was achieved using a 4-Fr RC-type catheter (RC® 80 cm; Medikit) within a triple coaxial system (Carnelian® HF 2.7Fr., Carnelian® Marvel 1.9 Fr.; Tokai Medical Products, Aichi, Japan), and a 0.016-inch guidewire (ASAHI Meister®, ASAHI INTECC, Nagoya, Japan). Embolization was performed using the following coils: Azur Soft 3D® (Terumo, Tokyo, Japan): 1.5 mm × 4 cm, 2 mm × 6 cm, 2 mm × 3 cm, 3 mm × 10 cm and Target® coil (Japan Stryker Inc., Tokyo, Japan): 1.5 mm × 3 cm, 2 mm × 3 cm (x2), 2 mm × 4 cm. Postembolization angiography confirmed distal vessel patency via collateral circulation through the arcade. The patient’s postembolization course was uneventful, with no signs of bowel ischemia.

The oral and maxillofacial surgery team performed hemostasis and suturing of the patient’s facial lacerations. During concurrent interventional radiology and oral surgery procedures, the patient experienced respiratory and circulatory instability, requiring intubation, vasopressor support, and intensive care unit admission.

In addition to mesenteric and retroperitoneal hemorrhage, the patient experienced significant facial bleeding, requiring massive fluid resuscitation and a total transfusion of 20 units of fresh frozen plasma and 6 units of red blood cells.

Follow-up CT performed on day 5 after injury revealed no evidence of intestinal blood flow disturbance or pseudoaneurysm. The patient’s general condition gradually improved, and he began enteral nutrition on day 7 after injury. After treatment, the patient recovered well and was eventually discharged. There was no recurrence of abdominal symptoms or intestinal ischemia at the 1-year follow-up appointment.

## Discussion

Mesenteric injuries are rare, accounting for approximately 1% of blunt abdominal trauma cases [[1], [2], [3], [4], [5], [6], [7], [8]]. They often result from rapid deceleration forces, with seatbelt compression being a common mechanism [2]. This case highlights a unique mechanism of injury involving a detached tire. The patient presented with closed mesenteric hematoma, a rare condition that can be challenging to manage due to its potential for delayed bowel ischemia and other complications.

Early diagnosis with CT imaging and prompt intervention are crucial in managing mesenteric injuries. TAE is a valuable minimally invasive treatment option for hemodynamically stable patients with ongoing bleeding. In this case, TAE successfully controlled the bleeding and prevented bowel ischemia, thereby avoiding the need for more invasive surgery.

Herein, we report the case of a 70-year-old man who presented with closed mesenteric hematoma following blunt abdominal trauma from a detached tire. Mesenteric hematomas can lead to bowel ischemia, necrosis, peritonitis, and sepsis, emphasizing the need for early diagnosis and effective management.

In this case, the first abdominal CT scan revealed mesenteric hematoma. Angiography confirmed vascular wall injury of a branch of the ileocolic artery, and TAE was successfully performed. TAE effectively controlled bleeding and prevented bowel ischemia, preventing the need for more invasive surgery.

Management of mesenteric injuries can involve surgical intervention, interventional radiology (IVR) with embolization, or conservative treatment approaches. The treatment choice depends on several factors, including hemodynamic stability, the presence of associated injuries, and the extent of bowel ischemia. IVR with embolization has gained increasing recognition as a safe, effective, and minimally invasive treatment option for hemodynamically stable patients with isolated mesenteric injuries and persistent active bleeding from a mesenteric artery [[9], [10], [11]].

Microcoils are the most commonly used embolic agents, although other materials such as N-butyl-2-cyanoacrylate (NBCA) may also be used [[10], [11]]. Super-selective embolization is safe and effective; however, the potential for extensive bowel ischemia must be carefully considered if a major vessel is embolized. Additionally, there is a risk of postembolization rebleeding due to collateral blood flow, particularly if only the injured segment is embolized.

This case highlights the successful use of TAE for managing closed mesenteric hematoma following blunt trauma. In patients with severe multiple traumas, the patient’s overall condition, location and severity of injuries, and prioritization of treatment must be carefully considered to ensure the proper sequence and modality of interventions.

In conclusion, this case illustrates the successful use of TAE for the management of isolated mesenteric hematoma following blunt trauma. Selective TAE is a safe, effective, and minimally invasive technique for controlling bleeding and preventing complications in carefully selected patients with mesenteric injuries.

## Patient consent

Informed consent was obtained for the publication of this case report.
